# Critical complexity in environmental health practice: simplify and complexify

**DOI:** 10.1186/1476-069X-11-S1-S19

**Published:** 2012-06-28

**Authors:** Hans Keune

**Affiliations:** 1 Research Institute for Nature and Forest (INBO), Brussels; Centre of Expertise for Environment and Health, Faculty of Political and Social Sciences, University of Antwerp; naXys, Namur Center for Complex Systems, University of Namur, Belgium

## Abstract

The magic word ‘complexity’ has been buzzing around in science, policy and society for quite some time now. There seems to be a common feel for a ‘new way’ of doing things, for overcoming the limits of tradition. From the combined perspective of critical complexity thinking and environment and health practice we want to contribute to the development of alternative routines that may help overcome the limitations of traditional environment and health science. On the one hand traditional environment and health science is too self-confident with respect to potential scientific insight in environment and health problems: complexity condemns us to limited and ambiguous knowledge and the need for simplification. A more modest attitude would be more realistic from that point of view. On the other hand from a problem solving perspective more boldness is required. Waiting for Godot (perfect undisputed knowledge) will not help us with respect to the challenges posed to society by environment and health problems. A sense of urgency is legitimate: the paralysis by traditional analysis should be resolved. Nevertheless this sense of urgency should not withhold us from investing in the problem solving quality of our endeavour; quality takes time, fastness from a quality perspective often leads us to a standstill. We propose the concept of critical complexification of environment and health practice that will enable the integration of relevant actors and factors in a pragmatic manner. We will illustrate this with practical examples and especially draw attention to the practical complexities involved, confronting us not only with fundamental questions, but also with fundamental challenges.

## Introduction

The magic word ‘complexity’ has been buzzing around in science, policy and society for quite some time now (for recent examples [1,2]). There seems to be a common feel for a ‘new way’ of doing things, for overcoming the limits of tradition. The ‘new way’ though is conceived radically differently by two important schools of complexity thinking. Whereas the Santa Fé school [3,4] merely believes that new scientific strategies in the face of complexity in the end will bring us closer to the modern aim of ever more perfect knowledge and control, the critical complexity school [5-7] points out that limits of knowledge are inherent to complexity, necessitating reduction and critical reflection on the normative basis for any simplification. The intense debates about complexity seem to be mainly located in the salons and saloons of scientific and social debate, focussing not so much on ‘how to actually do it’ and with little reflection on practical experiences. We will focus on the art of complexity and apply this to one of the most challenging and complex fields of today: the relationship between environment and health. Grand old men in the field of environment and health, Philippe Grandjean [8,9], David Briggs [10] and David Gee [11], critically reflect on the limits of current environment and health science when judged from a problem solving perspective and warn us of the lack of relevance of current scientific practice with respect to complex reality. From the combined perspective of critical complexity thinking and environment and health practise we want to contribute to the development of alternative routines that may help overcome the limitations of traditional environment and health science. We will especially draw attention to the practical complexities involved, confronting us not only with fundamental questions, but also with fundamental challenges.

## Review

### Critical complexity

#### Complexity

Most environment and health experts will probably agree that the field of environment and health is a characteristic example of complexity: the interaction of all relevant elements, both pollutants and health parameters as well as a wide range of intervening variables such as lifestyle and genetic factors create a very complex interplay that is hardly possible to conceive in all its complexity, let alone fully measure, describe and comprehend. The study of cocktail effects of different chemical agents in interaction with each other and human health is a good example of the natural scientific complexity of environment and health issues. It is recognized to be of the utmost importance for a more realistic view of the field for some time [12], but cocktail effects only seem to be receiving considerable scientific and policy attention in recent years [13]. Moreover it is recognized more and more that the social complexity of environment and health should also be taken into account if we want to create problem solving horizons: social dynamics, both in science, politics and society as a whole, form an integral part of environment and health issues. The growing awareness in the field of climate change that social scientific expertise is essential in order to better deal with the challenges posed on our society by climate change [14,15] is exemplary in this respect. For a more extensive account on natural scientific and social scientific complexity with respect to environment and health see e.g. Keune et al. [16]. We will now focus on critical complexity.

#### Critical complexity

The study of cocktail effects is a good example of a different view on complexity in that environment and health issues are no longer reduced to studying single pollutants and their individual potential health effects. Recent insights show that cocktails of pollutants create dynamics surpassing the level of effect of single pollutants and may result in stronger combined health effects than would be expected when simply adding the single pollutants’ effects [13]. This means that current mainstream environment and health knowledge does not suffice as a basis for evidence based policy making. We can no longer conclude that safety is assured when individual levels of pollutants are below specific individual thresholds that are believed to be safe. Nevertheless, even though strategies taking into account a bigger and more complex picture of reality by being less reductionist are new in their focus and method, they may remain traditional in their promise of perfect and undisputable knowledge, and may remain traditional in their scientific analysis. The study of cocktail effects may be an example of this. A critical view on complexity challenges our potential knowledge of complex phenomena, stating that because of the intrinsic properties of complexity we are condemned to limited knowledge of complexity. We will not present a complete overview of properties of complexity [5,17] here, we will only refer to some properties so as to illustrate some of the problems of dealing with complexity. One important property of complexity is emergence [17]: the presence of a great number of (often simple) system components that interact in a manner that cannot be explained by the characteristics of the individual components. Another important feature of complexity is non-linearity [17]: due to partly non-linear input - output functions, complex systems will show unpredictable behaviour. Furthermore, complexity is to be characterized by temporality [18]: complex systems echo their history, their memory of the past in the present and future, be it in a selective and non-linear manner. Finally we mention the problematic issue of reduction [17]: any knowledge we have about a complex system is a reduction of its complexity.

We have to realize that the challenge of gaining knowledge about complexity will be as important as the challenge to act based on limited knowledge. Both challenges are interrelated. Cilliers [17]: “*More than one description of a complex system is possible. Different descriptions will decompose the system in different ways. Different descriptions may also have different degrees of complexity.”* At the very heart of a critical complexity perspective lie fundamental questions about the nature and status of meaningful knowledge, for which no unambiguous criteria exist (ibid.). The interpretative nature of knowledge is closely related to normative choices, ethical issues, and political issues. Cilliers [17] pleads for a modest attitude towards complexity and knowledge about complexity: “*knowledge is provisional. We cannot make purely objective and final claims about our complex world. We have to make choices and thus we cannot escape the normative or ethical domain*.” Philippe Grandjean [8] seems to agree: *“Risk assessment can never become completely objective”*. He is especially critical on environmental health-science from a problem solving perspective: due to complexities traditional science will not be fit to tackle environmental health-problems. *“Risk assessment must become less reductionist and less focused on obtaining complete information on all aspects of individual hazards. Statistical acceptance of the null hypothesis should never be interpreted as proof of safety.* (*…*) *Given that decisions will involve stakeholders*, *risk perception should receive increased attention as a crucial aspect that is not dependent on a formalized scheme of evaluation” *[8]. According to Grandjean, standard scientific approaches do not fully fit issues, in focus and method (too much focus on simplified models and effects of single hazards one by one) or in interpretation (too strict analytical standards). Grandjean stresses the need for a different scientific approach and application in policy practice of a precautionary principle. David Briggs [10] pleads for more integrated methods of assessment. Key challenges do not only relate to the content of analysis, environment and health-problems, but also to the involvement of relevant actor perspectives. With regard to complexity, Briggs (like Grandjean) criticizes traditional forms of assessment, and proposes to focus on a real world perspective in which the issue of problem framing becomes of main importance. The involvement of relevant actors according to Briggs need not be limited to scientists alone: the involvement of stakeholders is also important, at an early stage. According to Briggs, despite numerous pleas for and ambitious ideals with respect to environment and health, the application of integrated approaches in research practice is still in its infancy.

We may conclude here that traditional environment and health science is too self-confident with respect to potential scientific insight in environment and health problems. Environment and health complexity condemns us to limited and ambiguous knowledge. A more modest attitude would be more realistic from that point of view. From a problem solving perspective more boldness is required. Waiting for Godot (perfect undisputed knowledge) will not help us with respect to the challenges posed to society by environment and health problems. Instead of mainly focussing on what we don’t know yet we should focus more on what we already do know in order to facilitate more pragmatic problem solving interpretations of environment and health complexity. The traditional focus on strict statistical analysis of the tiny fragments of reality that we are able to measure is challenged by ethical concern from a societal problem solving perspective and demands critical qualitative reflection on the ambitions and responsibilities of environment and health science. A sense of urgency is legitimate: the paralysis by traditional analysis should be overcome. Simultaneously this sense of urgency should not withhold us from investing in the problem solving quality of our endeavour; quality takes time, fastness from a quality perspective often leads us to standstill [18].

#### Open choices, enabling limitations

Making choices is essential from a critical complexity perspective in two important respects. One is that we can never have perfect knowledge about complex issues: we choose our picture of reality but have to realize that each picture has limitations. This picture can have many forms, e.g. problem framing, a model, a research ambition, a policy action or public debate. Second, we cannot objectify which picture of complex reality is best or better than other pictures, thus knowledge will not be unambiguous. Does this mean that we should *not* reduce complexity in order to deal with it realistically and that we have to accept that in principal all knowledge is of equal significance? The answer to both questions is no. According to Cilliers [17]: *“Limited’ knowledge is not equivalent to ‘any’ knowledge. If this were so*, *any modest claim*, *i.e. any claim with some provisionality or qualification attached to it*, *would be relativistic.* (*…*) *Modest claims are not relativistic and*, *therefore*, *weak. They become an invitation to continue the process of generating understanding.”* And: *“This does not imply that we can know nothing about complex systems*, *or that the knowledge claims we make about them have to be vague*, *insipid or weak. We can make strong claims*, *but since these claims are limited*, *we have to be modest about them.”* In the process of knowledge generation we constantly have to make interpretive choices: *“Knowledge is interpreted data. This leads us to the next big question: what is involved in interpretation*, *and who* (*or what*) *can do it?” *[19].

By choosing our picture or reality, we draw boundaries. We draw ontological boundaries that frame the picture of complex reality: knowledge boundaries. And we draw epistemological boundaries with respect to the generation of knowledge on complexity: disciplinary and transdiciplinary boundaries. Moreover do we ‘perform’ ethics in our boundary work: we choose what we consider to be relevant, important, just, better, best. Ontologically the boundaries can be bold or modest, flexible or inflexible. Epistemologically the boundaries can be closed and inward looking or open and an invitation to dialogue with others and other forms of knowledge. Boundaries create a difference as they distinguish the inside of the picture of complex reality from the outside and distinguish one picture of complex reality from another picture. A picture of the health effects of one pollutant is different to a picture of the health effects of another pollutant, and is different to a picture of the health effects of a cocktail of pollutants. A natural scientific picture of environment and health will focus on other aspects than a social scientific picture, even when looking at the same environment and health issue. In fact scientists with similar disciplinary background will also potentially create completely different pictures of similar environment and health issues. A scientific picture will probably focus on other characteristics of complexity than a picture of policy makers or stakeholders. This does not necessarily mean that some pictures are better than others, nor does it necessarily mean that we should fuse all pictures into one super picture of complex reality. Different pictures may complement and may enrich each other, but may also criticize and compete with each other. The way we choose to deal with difference is of the utmost importance in the case of complexity [20]. We can consider openness to other, different pictures of complexity and other perspectives on complexity important as a test of one’s own picture: is our picture of complex reality robust when we compare it to other pictures, can we learn from other pictures and do we pass the test of being criticized by others about the robustness of our picture?

Theoretically this might imply that the more different viewpoints we take on board and the more critical mass we organize to test our endeavour, the more robust our end product, be it knowledge, be it (e.g. policy) action, will be. This would indeed connect well to the ideal of integrated assessment proposed to us by Briggs [10] and the involvement of stakeholders proposed both by Briggs and Grandjean [8]. In fact we may broaden the basis of support for this openness with reference to other approaches in the familiar fields of risk governance and environmental science and policymaking that promote an ‘open arms approach’, such as the analytical-deliberative approach [21] and the extended peer review approach [22]. Cilliers [5] proposes this theoretical ideal of openness to and respect for differences as an ethics of complexity. Cilliers [23] nuances the ideal by pointing at the notion of power: *“The argument from complexity claims that a single story*, *or in the words of Lyotard*, *a ‘coherent meta-narrative’ cannot describe any social system fully…The reason why a certain description is acceptable has to do less with rationality and more with power. We do not have to look hard to find examples of master-narratives which oppressed the ‘other’ in the system*, *whether they be of a different race*, *religion*, *gender or sexual orientation.”* Kunneman [7]: “(*…*) *difficulties become visible when we pose the question why we should prefer his ethics of differences above - for example - an ethics of care*, *or the discourse ethics propagated by Jurgen Habermas* (*…*) *or for that matter*, *the aggressively ‘masculine’ ethics connected with the Hip-Hop scene*, *or the ‘tribal’ ethics practiced with great brutality and with great economic success by Italian Mafia-families?*” We therefore do not want to proclaim a critical complexity perspective (whatever it would mean in practice) as just because of the intrinsic qualities of complexity, but merely propose it as a worthwhile companion when we picture complex reality. We propose to take the openness to and respect for differences as an ambition that is worthwhile testing, but consider it not to be immune to one of the most important ingredients of critical complexity: critical reflection. An intriguing example of the need for reflection on openness is the growing influence of industry experts in important policy advisory expert panels over the last decades [24-26], of which the International Agency for Research on Cancer (IARC) is an important environment and health example. The IARC is part of the World Health Organisation and its mission is to coordinate and conduct research on the causes of human cancer, the mechanisms of carcinogenesis, and to develop scientific strategies for cancer control. Huff [24], a former Chief of the Unit responsible for the IARC Monographs, wrote about the unprecedented and growing industry influence on the Monographs. In the case of chemical exposures, this resulted in a lower risk evaluation for chemicals. And this leads us to the conclusion that openness is an ethical issue in itself.

The critical view on complexity is very important in better understanding and discussing the challenges posed by complexity. The critique unmasks weak spots in our understanding of and dealing with complexity. Moreover current critical complexity thinking may inspire to create alternatives routines of understanding of and dealing with complexity. We have to open up and narrow down simultaneously: we have to be more realistic in our reduction; we have to outsmart our limitations. Not by more of the same, but by differentiation. We should not though remain too much on an ideal theoretical level: we need to take into account practicalities, we have to be pragmatic. We have to find a clever balance between respect of complex reality and practical attainability: we have to be both informative and performative. As none of these aspects, choices and strategies can be objectified because of limited knowledge and ambiguity, we cannot refrain from ethics, otherwise we will be either lost in blindness or limitlessness and in fact get nowhere. We have to make conscious choices by asking ourselves what is important, what is relevant, what is the meaning of what we do.

### Critical complexification

*Critical complexification* means opening up boundaries that limit our view on complexity, connecting relevant contexts that will enrich our view and will enrich relevant contexts. Simultaneously, *critical complexification* has to set its own boundaries; otherwise nothing will happen, except staring at outer space forever with the friends we gather. Such boundaries will be different in character though than turning ones back to others, to whom and what are excluded. The boundaries are always open for critique, for discussion, for reflexion. *Critical complexification* also means challenge: we challenge complex reality, we challenge ourselves, we challenge others. We also challenge ‘our’ or ‘their’ current practice of dealing with complexity. We challenge the actors, we challenge the contexts of those actors, we challenge their knowledge, we challenge their practice. Challenge means critique in a constructive manner. It not only means to ask fundamental and radical questions about what others do and know and by what motives, it also means to invite, cooperate, enable, enrich in order to better deal with complexity. And last but not least, through *critical complexification* we face the challenges practice will have in store for us.

We will use the term *critical complexification* to describe the process of critically dealing with complexity in practice. *Complexification* draws our attention to our selection of relevant of elements of complexity that we want to take on board when picturing complexity in order to do justice both to complex reality and to the ambition(s) we choose with respect to dealing with complex reality. The term *complexification* was used before by other authors in a more or less similar fashion [27,28]. Next to this we mean it also to be a word of action, drawing attention to the practical aspects of the art of complexity: how do we *complexify*? *Critical* draws our attention to critical reflection on our ambitions and actions and to challenging the quality of our activities and outputs. On the one hand this draws our attention to the need to reflect on our choices from an ethical perspective: what is the justification for our ambitions and our actions? On the other hand this draws our attention to the issue of critical mass: what is the basis for challenging the quality of our ambitions and actions, or in other words, which assessment criteria are relevant and who should be involved in the assessment?

In discussing practical elements of *critical complexification* we will refer to two practical contexts in which difference (and diversity) was considered relevant to dealing with the complexity of environment and health and was approached differently than in mainstream environment and health science and policy making. We invite those readers who want to learn more, to read the references, and only introduce the cases very briefly.

#### Case analytical deliberative approach (AD)

Instigated by policy representatives together with medical and environmental scientific experts and policymakers, in Flanders (Belgium) an *action-plan* was developed for setting policy priorities with regard to human bio-monitoring results: from research results to policy action [29]. The *action-plan* was inspired by the analytical deliberative approach [21], an approach that combines scientific complexity and social complexity by linking expert debate with social debate. In the practice of the *action-plan* it concerned close interdisciplinary cooperation: the general approach had to be negotiated between totally different disciplinary backgrounds and natural and social scientific data were combined. It also concerned close cooperation with policy representatives: the research had to be policy relevant, which puts totally different demands on research than just scientific ones. Furthermore, both experts and stakeholders were involved. The basic problem that needed to be solved was choosing between policy options that are rather different in nature, e.g. policy on asthma incidence and policy on pollution from pesticides. The choice is based on different assessment criteria: seriousness of health risks, policy aspects and social aspects. The procedure was organised as follows: first desk research provides the different options with background information concerning the different assessment criteria. The environmental and health information relevant to assess the health risk is being gathered by natural scientists. The social scientists are responsible for policy-related and social aspects. Second, the desk research information is assessed in an expert consultation. Experts with regard to environment and health assess the health risk criterion, policy experts the policy aspects as do social experts the social aspects. These assessments result in both *quantitative information* (priority rankings of options on different criteria) and *qualitative information* (arguments, difference of opinion, uncertainties). The outcomes of the expert consultation are processed in a multi-criteria analysis [30,31] as well as in an account of (other) qualifications. Third the results of both desk research and expert consultation are discussed by a stakeholder jury that gives advice on the basis of all information: different from experts a societal view deals with the political question of deciding what’s important considering all aspects. Finally the procedure is aimed at a well informed and substantiated decision-making by the policymakers. In the following we will call this the AD-case.

#### Case expert elicitation (EE)

The EU HENVINET project had the ambition to synthesize scientific information available on a number of topics of high relevance to policy makers in environment and health [32]: brominated flame retardants, phthalates, the impacts of climate change on asthma and other respiratory disorders, the influence of environment health stressors on cancer induction, the pesticide CPF and nano particles. At first it was the ambition to focus mainly on the state of the art scientific knowledge, with a special interest in gaps of knowledge. By means of expert elicitation the gaps of knowledge were highlighted by using confidence levels for assessment of current scientific knowledge. During the work in progress a complementary focus developed through interdisciplinary reflections. By extending the horizon of the endeavour from only science to the problem solving policy perspective, the ambition was complemented by interpreting the synthesized available knowledge from a policy perspective, addressing the question which kind of policy action experts consider to be justifiable based on the identified state of scientific knowledge. As such the expert elicitation approach became helpful in overcoming the policy action impasse caused by the mere scientific knowledge oriented strategy for dealing with limited knowledge on complex issues. It did so by constructively discussing the weight of existing knowledge for potential policy action, thus stressing more the societal importance of the issues under study and considering to take action, rather than merely betting on the scientific quest for ever more knowledge. Both parts of the expert elicitation, the assessment of state of the art scientific knowledge by means of confidence levels and the problem solving interpretation by means of a qualitative questionnaire and a workshop discussion, were quite challenging for all experts involved, as it did not relate easily to mainstream environment and health scientific practice. In the following we will call this the EE-case.

### Practice

#### Embrace and structure

Important choices that have to be made when dealing with complexity concern relevant elements of complexity to take on board: which actors and factors are considered relevant? Who and what do we embrace and who are we? Part of the answer is in complex reality: reality poses specific challenges. Part of the answer is in our ambition with respect to reality: what do we hope to achieve? Creating knowledge as such is another challenge than creating policy relevant knowledge and is another ambition than developing problem solving actions. Focussing on individual pollutants poses another challenge than focussing on cocktails. And part of the answer is in the discussion amongst those who are in the driving seat: the cocktail of actors involved will create specific dynamics affecting the process. In the AD-case the team consisted of natural and social scientists and policy representatives. Amongst the actors consulted in the process were other natural and social scientists and policy representatives, and policy experts and stakeholders. Next to environment and health factors, policy and social factors were also taken into account. The ambition of the transdisciplinary team in the AD-case thus was clearly one of open arms, embracing a broad diversity of actors and factors. Moreover the ambition stretched the horizon of scientific research to concrete policy action plans. In the EE-case the ambition of the interdisciplinary team seemed largely limited to science: scientific experts assessing state of the art scientific insights. Nevertheless, initially the knowledge produced in the process was intended to be used in a policy context, rather than in a research context. The horizon in this case thus was also broadened from science to policy. Moreover difference of opinion amongst experts was considered potentially valuable information for policy makers, thus opening the visor to diversity of viewpoints.

Embracing relevant actors and factors cannot do without a procedural and structural way of working: structuring interaction and content and as such complexity. Without structure one runs the risk of endless research and discussion. In the AD-case a practice cycle was developed in which the process was streamlined from organisation of the procedure to the final choice of policy priorities: which actors were supposed to play which role, in which phase and based on which factors. Moreover as an analytical red thread a multi criteria analysis was used by which the diversity of (to a large extent incommensurable) information and opinions could be both embraced and structured so as to fit next steps in the process. In the EE-case the use of confidence levels by means of an online questionnaire initially formed the structuring backbone of the approach.

#### Historical identities and the art of negotiation

An openness to and respect for differences and diversity by embracing critical mass in the process of *critical complexification* has to take into account that actors involved have different identities. Identity is to a large extent determined by the social context, be it professional, be it private. In both the AD-case and the EE-case the professional background played important roles. As Ulanowicz [33] points out that systems differ according to their history, so do professional contexts differ in professional tradition. Obvious examples are differences between quantitative and qualitative scientific approaches, between a focus on knowledge and a focus on action, between natural sciences and social sciences, between science and policy. The teams cooperating in both cases had to undertake a lot of negotiation during the process, the importance of which is often underestimated both in terms of impact on the process and its output, but also in practical complexity. The richness of dialogue can be very beneficial to a broader and more integrated view on complexity, but it is not always easy. The mindsets of actors from specific contexts remain largely influenced by and focussed on their home-base contexts, and only to a lesser extent to the new joint context. This is beneficial from the point of view of specific expertise, and this is needed. But it can become problematic in the perception of other expert contexts: one is full of one’s own expertise and related complexity, and has only limited sight of the complexity of other expertise, and in fact often underestimates this. This to a large extent cannot be avoided, as experts are often overloaded with complexity from their own context and are constantly attracted by context specific interests, rewards, challenges. This also means that the openness towards other forms of expertise is limited, as they only have limited attention for it and only limited interest. The transferability of expertise from one context to the other is possible of course, but will be more difficult once experts’ contexts differ more. This poses the question whether we should invest in transfer of context specific expert knowledge to other expert contexts, or that we should focus on cooperation in well balanced inter- and transdisciplinary teams. From the experience of the social scientific contribution in both cases it can be concluded that teamwork currently is absolutely necessary. Even after years of intense cooperation, natural scientific colleagues often still do not have clear sight of the complexity social science deals with. This would make a plea for constant and direct involvement of social scientists and in fact to the notion of the old saying: ‘Let the cobbler stick to his last’. This also holds true for transdisciplinary cooperation between scientists and policy makers.

In the EE-case there was intense debate on whether the ‘I don’t know option’ should be included in the questionnaire for the experts that were to be consulted on their confidence in the state of the art of science. Opponents mainly worried about low response rates and thus mainly took a quantitative perspective on this: the ‘I don’t know option’ would provide the consulted scientists an easy way out of difficult questions, thus lowering the response rate for specific questions. Proponents stated the ‘I don’t know option’ to be important from a qualitative perspective: it would allow analysts to know better if they measured knowledgeable answers or forced and perhaps partly unknowledgeable answers. The proponents considered environment and health issues too complex to expect all scientists to know enough about all relevant aspects in the causal chain from exposure to health effect that was to be addressed. In the end it was decided not to take up the ‘I don’t know option’, thus the ‘quantitative camp’ won. Afterwards though, some scientists that were consulted in the expert elicitation said they sometimes felt rather uncomfortable due to absence of this option. This example shows how different scientific backgrounds may have completely different perceptions of research quality. When cooperating, they do not always find it easy to reach consensus, and in fact, in this case, it was impossible: both options excluded each other.

#### Ambition dynamics

In the AD-case elements of *critical complexification* were introduced by the social scientists: introducing other relevant actors/factors and critical reflection from a problem solving perspective. These aspects were relatively easily agreed upon by the natural scientists and policy makers. Trying to bring ambitions into practice however creates new dynamics that may cause a boomerang effect. Once the application in practice creates pressure on their work (e.g. time pressure, pressure on their role as experts, practical pressure by complicating their own or the joint effort) the enthusiasm of natural scientists and policy makers often was overshadowed by concern for practical and analytical constraints. The ambitions thus are not necessarily stable: developments were never linear or predictable or in one direction and ambitions may always be disputed. The dynamics of ambitions in practice may also take another turn though in that new developments in practice may stimulate ambitions that support a *critical complexification*. In the EE-case initially it was the ambition to encompass all aspects from pollution to health impact, including societal impact. In practice nevertheless the societal aspects hardly got any attention. At a later stage due to interdisciplinary reflections on this gap, some of these aspects were touched upon by integrating a problem solving perspective. Ambitions as such also can have a stimulating impact on a *critical complexification* of practice and thus are strategic in this respect.

#### Complexifiers: Trojan horses and other strategies

An essential element of the *critical complexification* of practice was a strategic way of working. An important strategic move in the first stages of the AD-case (the conceptual design phase) that proved to be of decisive importance was an active listening approach: the use of an internal reflective questionnaire. At first the practical relevance of *critical complexification* as such proved difficult to agree upon by the colleagues from natural science and policymaking. However, when elements of *critical complexification* were presented by means of open questions in an in-group questionnaire (who are relevant actors and factors?), based on the group results these elements gained support. In fact, it led to a breakthrough in the conceptual development process and formed the basis for the practice cycle in which questions of openness to relevant actors and factors were pragmatically dealt with. As such the internal reflective questionnaire can be seen as a *complexifier*: an element that will have a catalyst effect on the process of *critical complexification*.

In the EE-case the problem solving turn from mainly focussing on overcoming gaps in science to overcoming gaps between science and policymaking was triggered by using references to ambitions as *complexifiers*. The social scientist involved in the project while trying to introduce a *critical complexification* perspective, realized it was not easy to convince the principal coordinator of the EE-case. The potential benefits of *critical complexification* were countered by pointing out practical complexities that would put further pressure on what in itself was already quite a challenging pioneering endeavour, let alone put pressure on the loyalty to the expert elicitation project of the natural scientists in the team. The social scientist used reference to ambitions that were part of the initial project aims, be it mainly dormant, and ambitions from the professional background of the principal coordinator of the project as *complexifiers*. He pointed out the initial ambition of policy relevance of the project as an argument for integrating a problem solving perspective. Also he referred to two grand old men in the field of environment and health for whom he knew the coordinator had high respect, and who promote a problem solving turn in the field of environment and health [8,9,11]. Being part of the project one of them in fact had criticized the absence of a clear problem solving perspective in the early phases of the project. The fact that idealistic ambitions are often not easily applied in practice thus does not withhold them from being used as *complexifiers*: from a dormant or Ten Commandments’ status to becoming seeds of practical change and inspiration. Apart from being an example of how ambitions can be *complexifiers*, the EE-case example also exemplifies how an outsider perspective can function as *complexifier*: the social scientist joined the project at a later stage, thus as a newcomer could reflect on the work in progress from some distance. Another example of the strategic impact of outsider perspectives is the use of external (outside of the team) feedback on the process. In the AD-case all external actors contributing to the project were asked for their feedback on the project. The vast majority evaluate openness to outsider perspectives and diversity of actors to be worthwhile. This is of course a bonus for those organizing such processes and for the end-user of the outcomes (e.g. policymakers). Simultaneously this can be perceived both as a stimulus and a pressure for prolonging such openness.

Experience from the AD-case shows that negative connotations may also be the result of strategic behaviour, resulting in what we in retrospect may characterize as a *Trojan horse strategy*. By joining conceptual discussions on policy interpretation of scientific research outcomes and reflecting on the ambitions of both natural scientists and policy representatives step by step from an active listening approach the role of the social scientist evolved to one of more central importance. The characterization ‘Trojan horse’ is mirrored in the expression of one of the senior natural scientists involved, saying she (on the level of ambition) approved of the social scientific contribution (which is in the AD-case in fact one of *critical complexification*, and as such is a *complexifier*), but she sometimes felt like an object of some social scientific experiment. Colleagues with natural scientific background sometimes react as if they feel lured into unexpected complexity, unknown to their expertise, difficult to handle and sometimes confrontational, and they either question its usefulness or appear to be unable to articulate the benefits themselves. This is also reflected in the often heard concern of the natural scientists and their counterparts in policy making that the *complexifying* approach is relevant and interesting but should not stand in the way of the research or policy agenda and should not complicate the already complicated research and policy endeavour. In the section on quality (see below) we will return to this issue. First we focus on methodological aspects of *critical complexification*.

#### Method: path finding

According to Morin [6]* ‘the method emerges from the research’*. Here the word method is used in its original meaning as path, indicating that only in travelling the right method appears. This connects well to learning by doing and negotiation, as well as with the diversity of relevant elements of complexity taken into account in *critical complexification*. This does not mean that practice is sacred and methodologies and reflections from methodological expert debate are only of secondary importance. It means that they complement each other so as to serve the ambitions chosen for the endeavour and the challenges posed by practice along the way. The nature of complexity moreover challenges what we might call textbook approaches of strict and unambiguous application of methods, almost as if they should be applied regardless of complexities, of that which cannot be captured, controlled or foreseen completely. With respect to method and complexity the distinguished methodological thinker Patton [34] refers to the following metaphor used by Gleick [35] to explain the very nature of inquiry into chaos: *“It’s like walking through a maze whose walls rearrange themselves with every step you take”*.

In the *critical complexification* of practice, dealing with unforeseen complexities and imperfections poses important challenges. Flexibility is essential: the need for context specific manoeuvre also from a methodological point of view. In fact, to a large extent methodological developments are part of the process and contradict the usefulness of a Bible belt approach of strict application of rigour. Moreover flexibility shows in a pluralist approach of using a diversity of methodological concepts whenever considered appropriate: e.g. a diversity of participatory approaches (e.g. the analytical deliberative approach, extended peer review, expert elicitation and participatory evaluation) and analytical approaches (e.g. multi-criteria analysis and qualitative analysis). The concepts of mixed methods and triangulation provide a conceptual basis for this eclectic praxis. Compared to single approach designs, mixed methods research is better equipped for complexity and provides opportunities for presenting a wider range of divergent views [36]. Quantitative methods provide relatively standardized, efficient, amenable information, which can be easily summarized and analyzed. Qualitative methods add contextual and cultural dimensions, which deepen the study by providing more natural information. Combining these two can thus be considered a ‘third approach’ [37]. Triangulation has been broadly defined by Denzin [38] as *‘the combination of methodologies in the study of the same phenomenon’*, incorporating both quantitative and qualitative approaches. The concept of triangulation is helpful not so much as to increase the validity of our findings in a conventional, positivistic sense, but rather as a strategy that allows new and deeper dimensions to emerge. One might get a fuller picture, but not a more ‘objective’ one [39]. Triangulation facilitates more in-depth-understanding in that it can capture a more complete, holistic, and contextual interpretation of the complex relation between environment and health within the complex social context of disciplines and stakeholders.

In the AD-case the practice cycle developed for the procedure of policy interpretation of research results is an example of this eclectic praxis within the general framework of an analytical deliberative approach: it combines several methodological elements within one process, in which both quantitative and qualitative data and assessment play a role, both expert elicitation and stakeholder consultation, and in which a diversity of relevant actors and factors is combined with multi-criteria and qualitative analysis. The EE-case also exemplifies the use of a diversity of methods: a mainly quantitative questionnaire with confidence levels on state of the art science, a mainly qualitative questionnaire with respect to the weight of knowledge for policy action and an expert workshop based on the outcomes of both questionnaires.

#### Quality: challenges and balances

Dealing with complex issues per definition bears the burden of imperfection. Whatever comforting concepts may promise, real life complexity will take its messy toll once travelling from conceptual ambition to real life practice. Practice is messy and stubborn and the scientific method incapable of total control. Moreover conflicting scientific standards and traditions may pose insurmountable ambiguities. A challenging issue in this respect is quality: how can we assess the quality of important but imperfect information. How can we assess the quality of a process of *critical complexification*? How can we balance ambition, importance, practicalities and imperfection? With respect to evaluation of analytical deliberative (or likewise) participatory processes objectifying quality criteria is considered to be very difficult. Renn and Schweizer [40] point out that the diversity of concepts and background philosophies is one of the reasons for this. Rowe et al. [41] conclude that the complexity of participatory processes makes it difficult to identify clear benchmarks for evaluation. Rauschmayer et al. [42] stress the fact that such processes involve a diversity of actors, and as such a diversity of preferences, also from the point of view of process evaluation. This may lead to the fact that process outcomes are valued differently from different actor perspectives. They propose the use of participatory evaluation.

Processes of *critical complexification* have similar characteristics regarding quality assessment. Practical complexity illustrates how *critical complexification* cannot be judged unambiguously: the fact that practice of *critical complexification* is difficult can be seen positively as a necessary and bold challenge and negatively as an insurmountable obstacle or even a threat. On the one hand the ambition of *critical complexification* may be severely challenged by those who are taken by surprise by the (sometimes drastic and often underestimated) practical consequences for their own work and expert status. On the other hand, a positive effect of taking complexity on board is that this will enhance the realistic character and better facilitate a problem solving perspective. Moreover it may be the only way to deal with complexity, implying that dealing with complexity and respecting complexity per definition will be practically complex, leaving no other alternative than leave it untouched. In fact, in the AD-case several participants in the process as well as some international experts reviewing the project, stated that it will lead to a more efficient translation of scientific knowledge in policy actions, thus can be seen as an investment in quality that will potentially have positive returns. As one of the policy representatives in the AD-case pointed out when reflecting on the rather complicated procedure being proposed in the beginning: “*It looks rather complex to me*, *but I cannot think of any alternative in order to better deal with the challenge* (HK: translating environment and health science into policy action) *ahead of us*”.

## Conclusions

We proposed the concept of *critical complexification* as a companion of alternative boldness: embracing complexity in a realistic and problem solving manner. Simultaneously we have to be pragmatic: we have to have the courage to make choices, thateven though imperfect, will open windows of opportunity of dealing with complexity in respect of both complexity and diversity of viewpoints on complexity. We cannot present a recipe for *critical complexification* or define it like a definition of the speed of light, of a ‘how to boil an egg’. Neither can we present an easy approach. Perhaps we best take *‘Zen and the Art of Motorcycle Maintenance: An Inquiry into Values’ *[43] as a source of inspiration for a combination of traditional and critical complexity science, and at minimum perceive is as an invitation for necessary dialogue and cooperation. Critical reflection on current environment and health science and policy is needed anyhow. Imagine a doctor (environment and health expert) and a patient (polluted society): should the doctor reside to individual ever more specialized diagnosis even though the patient shows serious health complications?

## List of abbreviations used

AD case: analytical deliberative approach case; EE case: expert elicitation case; HENVINET: Health and ENVIronment NETwork; IARC: International Agency for Research on Cancer

## Competing interests

The author declares that he has no competing interests.
